# A Polygenic Risk Analysis for Identifying Ulcerative Colitis Patients with European Ancestry

**DOI:** 10.3390/genes15060684

**Published:** 2024-05-25

**Authors:** Ling Liu, Yiming Wu, Yizhou Li, Menglong Li

**Affiliations:** 1 College of Chemistry, Sichuan University, Chengdu 610065, China; 2 College of Life Science, China West Normal University, Nanchong 637009, China; 3 College of Cyber Science and Engineering, Sichuan University, Chengdu 610065, China

**Keywords:** ulcerative colitis, polygenic risk score, single-nucleotide polymorphisms, population stratification

## Abstract

The incidence of ulcerative colitis (UC) has increased globally. As a complex disease, the genetic predisposition for UC could be estimated by the polygenic risk score (PRS), which aggregates the effects of a large number of genetic variants in a single quantity and shows promise in identifying individuals at higher lifetime risk of UC. Here, based on a cohort of 2869 UC cases and 2900 controls with genotype array datasets, we used PRSice-2 to calculate PRS, and systematically analyzed factors that could affect the power of PRS, including GWAS summary statistics, population stratification, and impact of variants. After leveraging a stepwise condition analysis, we eventually established the best PRS model, achieving an AUC of 0.713. Meanwhile, samples in the top 20% of the PRS distribution had a risk of UC more than ten times higher than samples in the lowest 20% (OR = 10.435, 95% CI 8.571–12.703). Our analyses demonstrated that including population-enriched, more disease-associated SNPs and using GWAS summary statistics from similar ethnic background can improve the power of PRS. Strictly following the principle of focusing on one population in all aspects of generating PRS can be a cost-effective way to apply genotype-array-derived PRS to practical risk estimation.

## 1. Introduction

Ulcerative colitis (UC), a major subtype of inflammatory bowel disease (IBD), is a complex disease characterized by chronic inflammation of the colon [1]. Although the precise pathogenesis of UC remains unknown, several factors, including genetic background, environmental factors, and mucosal immune dysregulation, have been proposed to contribute to its pathogenesis [1,2]. Given its high incidence in developed countries and the significant increase in incidence in developing countries [3,4], UC has evolved into a global burden with a significant impact on the patients’ quality of life as well as high costs for the health-care system [5]. Individuals predisposed to UC could benefit from a predictive model that alerts them to their disease risk and enables them to take early steps to reduce this risk. Given the stability of genetic markers, the polygenic risk score (PRS) derived from summary statistics of genome-wide association studies (GWAS) is a promising tool for inferring an individual’s genetic risk for complex diseases [6,7]. PRS has proven effective in predicting diseases such as prostate cancer and coronary heart disease [8,9,10]; however, its application in UC remains limited, which highlights substantial opportunities for further research. Specifically, refining PRS models to improve predictive accuracy could profoundly influence the clinical management strategies for UC. While PRS is a useful tool, the performance of the PRS obtained from different datasets varies due to a variety of factors. The classic PRS method calculates a score by summing up the risk alleles that an individual possesses, weighted by the risk allele effect sizes as estimated by a GWAS on the phenotype [11,12]. As a result, the single-nucleotide polymorphisms (SNPs) used and the selection of GWAS summary statistics are primary factors that influence the PRS performance. Additionally, PRS effectiveness also varies across populations due to differences in allele frequencies (AF) and linkage disequilibrium (LD) patterns, making ethnicity an important factor that may influence predictive ability [13,14]. Keeping the consistency between the base and target datasets usually yields better results. Although these factors are critical for the performance of PRS, some of them were unintentionally overlooked in previous studies; for example, the lack of population-specific GWAS summary or population confounding persists for the maximum sample size.

In this study, we analyzed a large cohort of UC cases and controls with genotype array datasets obtained from the Wellcome Trust Case Control Consortium 2 (WTCCC2). We refined the PRS model by progressively evaluating and optimizing conditions until the most effective model was achieved. Our optimal PRS model demonstrated a promising ability to distinguish UC cases from controls, achieving an AUC value of 0.713. Additionally, we thoroughly evaluated the various influencing factors that influence the performance of PRS across the procedures of generating the PRS, including the base and the target datasets. In conclusion, our study provides a comparative predictive model for identifying individuals at high risk of UC, and it may also help to the transferability of genetic risk estimators for the non-European populations.

## 2. Materials and Methods

### 2.1. Datasets

We obtained data from the WTCCC2 for 2869 UC cases and 2900 controls, the majority of whom are of European descent. DNA extracted from either blood or saliva samples was used to genotype UC cases with the Affymetrix GeneChip v6.0 and controls with the Illumina ImmunoBeadChip. The dataset included original files of genotype intensity as well as genotype calling results from standard genotype calling procedures. Only genotypes with a posterior probability greater than 0.9, as determined by the Chiamo algorithm, were included in this study and subjected to the QC procedure. Genotypes that failed to meet this threshold were designated as missing [15]. Finally, we received 932,533 and 196,524 SNPs for cases and controls on NCBI build 36 (also known as hg18), respectively.

### 2.2. Quality Control

We first used the liftOver tool to realign the genomic coordinates to the NCBI build 37 (also known as hg19) [16]. Pre-imputation quality control (QC) was implemented for the autosomal SNPs using Plink 1.90 [17], and SNPs were excluded if these met the following criteria: duplicated markers, call rate < 0.95 across all samples, minor allele frequency (MAF) < 0.01 and extreme deviation from Hardy–Weinberg equilibrium (*p* < 1.0 × 10^−6^ for controls and *p* < 1.0 × 10^−10^ for UC cases). Next, we used the snpflip to identify SNPs that needed to be flipped and subsequently flipped the SNPs on the reverse strand. The cryptic relations among the samples were inferred by conducting KING analysis; no related samples were identified with kinship exceeding 0.0884, representing a relationship of second-degree relatives or closer. Genotype array data were filtered sample-wise by exclusion on the basis of an insufficient call rate < 0.95, inconsistency between self-reported and genotype-measured sex, or excess heterozygosity rate (>mean ± 6 standard deviations) [18].

### 2.3. Identifying European Population from the Dataset

We performed admixture analyses to identify the European population from all samples [19]. A principal component analysis (PCA) plot was employed to validate the genetically identified European population. The population structure analyses and PCA were conducted based on the same set of variants filtered by the following process: merging all samples with all reference panels by Plink, then reducing LD between markers by removing all markers with r^2^ > 0.2 (--indep-pairwise 50 5 0.2), as well as markers in known high LD regions. Variants with MAF > 0.02 and call rate > 95% across the dataset (excluding A/T and C/G mutations) were retained. In population structure analyses, the participants were compared to European ancestry (EUR), East Asian (EAS), and Yoruba in Ibadan (YRI) from the 1000 Genomes Project (1KGP) database [20]; the parameter K was set to 3 in admixture analysis, and samples with a European fraction greater than or equal to 0.9 were deemed to be genetically identified European.

### 2.4. Genotype Imputation

Pre-phasing was performed before genotype imputation with SHAPEIT4 [21]. We adopted the default settings and used the corresponding genetic map files to run SHAPEIT4. The genotype imputation results were determined by the reference panel used, which can further affect the construction of the PRS. For comparison, we selected two reference panels to evaluate their impact on the PRS performance: a mixed-population reference panel consisting of 2504 samples from 1KGP Phase 3 (version 5b) and a European-population-specific reference panel consisting of 503 European samples from 1KGP Phase 3 (version 5b). Next, the Variant Call Format (VCF) files of the reference panels were converted to imp5 to run imputation. Then, imputation was performed using 5 Mb regions across the whole genome using IMPUTE5 with default parameters [22,23]. Post-imputation quality control has been conducted by removing SNPs with INFO score lower than 0.8. For consistency between the cases and controls, SNPs existing in both cases and controls were retained for PRS calculations. All analyses were restricted to SNPs on autosomal chromosomes.

### 2.5. GWAS Summary Statistics of UC

Two UC GWAS summary statistics were obtained from the NHGRI-EBI GWAS Catalog [24]. These summary statistics are the results of large IBD cohort analyses conducted by Liu JZ et al. [25] and de Lange KM et al. [26], with neither study including any samples from WTCCC2. Liu JZ et al.’s GWAS summary statistics were downloaded according to the study ID: GCST003045. This UC GWAS was conducted among 27,432 individuals of European ancestry, and summary statistics included information on 156,115 SNPs. The other summary statistics were downloaded by study ID: GCST004133. This was a GWAS for UC conducted using 45,975 participants of mixed ancestries, which provides summary statistics for 9,588,017 SNPs.

### 2.6. PRS Calculation

The PRS was calculated using PRSice-2, which is an efficient program for automating and simplifying PRS analyses on large-scale data [27]. It uses two datasets to calculate PRS: a base dataset (GWAS summary statistics) to derive the sets of SNPs and their effect size through clumping and thresholding steps based on an association *p*-value threshold; and a target dataset (our genetic data), from which the PRS is calculated based on SNP individual genotypes additively coded. For each set of SNPs, the score was then calculated according to Equation (1).

(1) $${PRS}_{j}=\frac{\sum S_{i}\times G_{ij}}{M_{j}}$$

where S_i_ is the summary statistic for the effective allele for the genotype i, G_ij_ is the genotype i for the j individual (coded as 0, 1, 2), and M_j_ is the number of alleles included in the PRS of the jth individual.

We utilized European population data from the 1KGP Phase 3 (version 5b) as the LD reference panel (r^2^ = 0.1) and obtained the PRS at a series of p-value thresholds (5 × 10^−8^, 5 × 10^−6^, 5 × 10^−4^, 0.05, 0.5 and 1). We then compared the different combinations of PRS calculations to explore the factors that influence PRS performance.

### 2.7. Population-Enriched Site Filtration

The gnomAD database curated allele frequencies of variants among different populations by summarizing 15,708 genomes [28]. For the aim of this study, we downloaded gnomAD v2.1.1 to extract the variants dominated by the European population. Specifically, the variants whose allele frequency in the European (non-Finnish) population (AF_nfe) was higher than the allele frequency (AF) in the general population were deemed the European population-enriched sites.

### 2.8. Statistical Analysis

To select the optimal PRS, we utilized standard logistic regression modeling to assess the predictive accuracy of PRS and each model incorporated a set of basic covariates, including sex and the top 10 principal components (PCs) of the genetic data. To prevent overfitting, we implemented 100 iterations of 5-fold cross-validation in our analysis. Discriminative accuracy was evaluated using the area under the receiver operating characteristic curve (AUC). The accuracy, sensitivity, specificity, positive predictive value (PPV) and negative predictive value (NPV) of the PRS models were also calculated. Unless otherwise specified, all reported evaluation metrics in the Results and Discussion sections are derived from the average of 100 iterations of 5-fold cross-validation.

(2) $$Accuracy=\frac{\left(TP+TN\right)}{\left(TP+FN+TN+FP\right)}$$

(3) $$Sensitivity=\frac{TP}{\left(TP+FN\right)}$$

(4) $$Specificity=\frac{TN}{\left(TN+FP\right)}$$

(5) $$PPV=\frac{TP}{\left(TP+FP\right)}$$

(6) $$NPV=\frac{TN}{\left(TN+FN\right)}$$

(7) $$AUC=\int_{x=0}^{1}TPR\left({FPR}^{-1}\left(x\right)\right)dx$$

**Figure 1:**
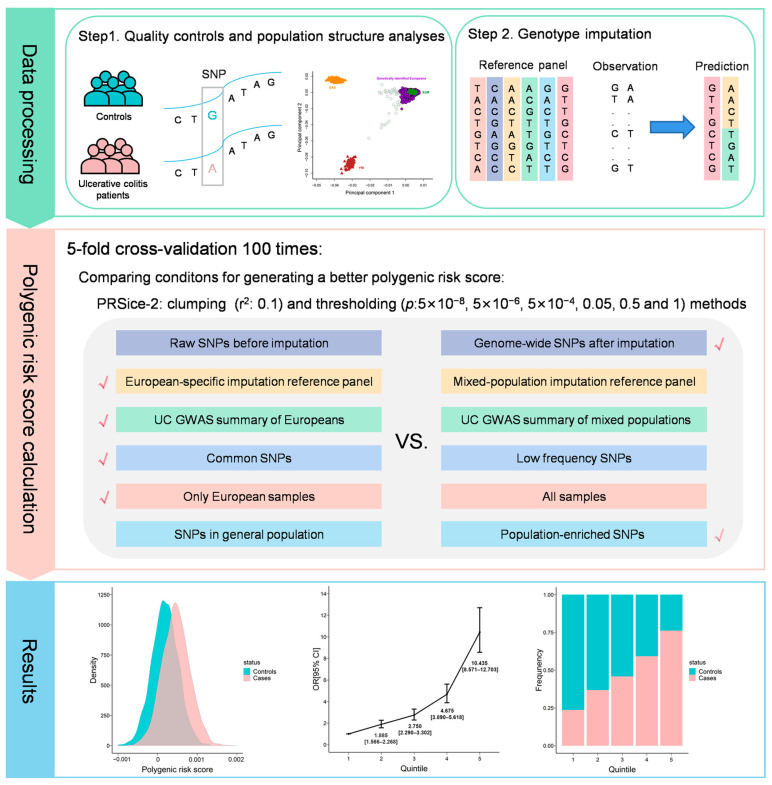
Overview of workflow. UC: ulcerative colitis, GWAS: genome-wide association study, SNPs: single-nucleotide polymorphisms, OR: odds ratio.

To evaluate the capability of the best PRS, which is a continuous score, we categorized the scores into quintiles and estimated the odds ratio (OR) of case–control status by contrasting each quintile to the lowest quintile in a logistic regression model. Sex and the top 10 PCs were included as covariates in the model. Statistical analyses were performed with R (v4.2.0) unless otherwise specified (Figure 1).

## 3. Results

### 3.1. Description of the Study Cohort

In total, 2781 UC cases and 2876 controls passed the QC procedure. After the implementation of a population identification procedure to select the European population, the numbers of cases and controls were reduced by 112 and 30 individuals, respectively (Figure S1). No gender bias was detected between the cases and controls (*p* > 0.05, assessed by Chi-square test) (Table 1). Information about the number of SNPs before and after genotype imputation is shown in Table 2.

### 3.2. Imputation Enhances the Power of PRS

**Figure 2:**
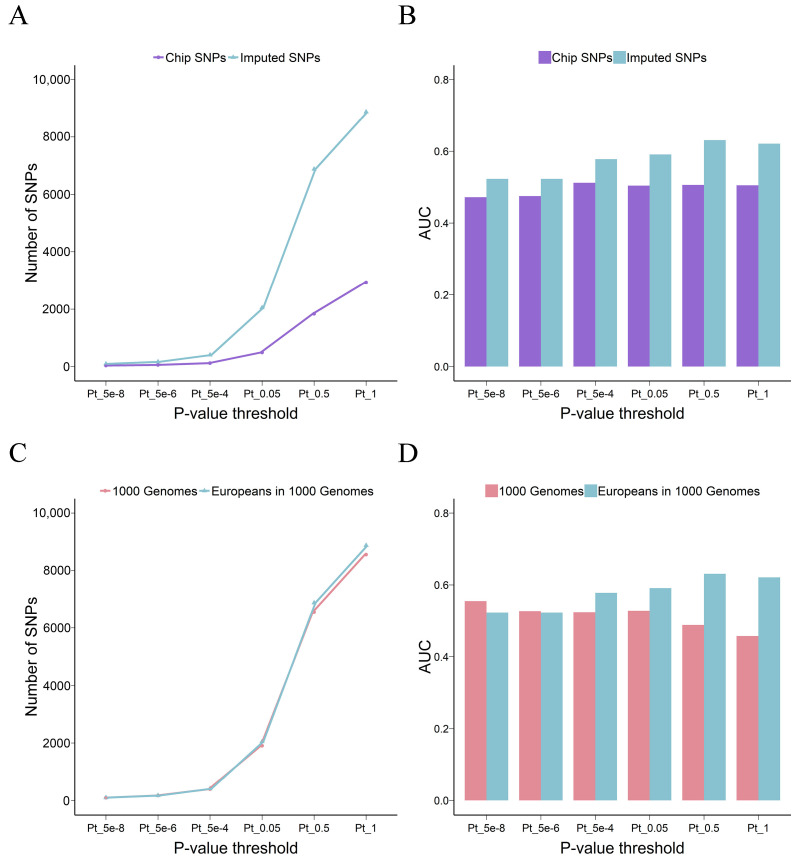
Effect of genotype imputation on the power of the PRS. Line plot shows the number of SNPs used to calculate PRSs and histogram shows the AUC values of the PRS model. (**A**,**B**) shows the results when comparing the chip SNPs and the imputed SNPs for the PRS calculation. (**C**,**D**) shows the results when comparing the different imputation reference panels. 1000 Genomes are the 1KGP Phase 3 (version 5b) reference panel, and Europeans in 1000 Genomes are the 503 European samples in 1KGP Phase 3 (version 5b) reference panel. PRS: polygenic risk score, SNPs: single-nucleotide polymorphisms, AUC: the area under the curve for the receiver operating characteristic curve.

The first study examined the impact of imputation on PRS using common SNPs (MAF > 0.05) and utilized de Lange KM et al.’s GWAS summary statistics. Using the imputed data to perform PRS analyses is particularly useful because imputation can enlarge the fraction of overlapping SNPs between the base GWAS and the target cohort (Figure 2A). Of the 14,289 post-QC and common SNPs on the chip, only 2930 were present in the GWAS summary statistics. The imputation process increased the number of overlapping SNPs to 8875 (Table S1).

There was evidence that PRS_imputed_ significantly outperformed PRS_chip_. PRS models trained on the imputed SNPs outperformed the models trained on the chip SNPs. The best AUC value for PRS_imputed_ was 0.631, while the best AUC value for PRS_chip_ was 0.512, and PRS_chip_ was less predictive than PRS_imputed_ across all *p*-value ranges (Figure 2B and Table S1). This is consistent with previous studies showing that the power of PRS can gradually increase with the addition of risk variants from the whole genome [6].

A critical step in genotype imputation is properly selecting the reference panel that contains haplotype patterns for the target datasets. The use of a population-specific reference panel has been proven to be important to improve the quality of genotype imputation [23]. Here, we further assessed how the choice of reference panel affects the predictive ability of PRS. As anticipated, the imputation result was better when the population-specific reference panel, specifically the European population from the 1KGP, was used (Figure S2 and Table 2). This further resulted in fewer SNPs used for PRS calculation when using the 1KGP reference panel imputation results (Figure 2C). More importantly, the overall performance of the PRS models was inferior when based on the imputation results from the 1KGP reference panel compared to those using the population-specific reference panel (Figure 2D).

### 3.3. Comparisons between GWAS Summary Statistics

GWAS summary statistics provide the corresponding effect size of SNPs for the PRS calculation, and selecting the best GWAS summary statistics plays a crucial and decisive role in the predictive power of the PRS model. Usually, a larger-scale GWAS can provide a more precise estimation of the effect size of SNPs for specific phenotypes; moreover, the effect size of SNPs for certain phenotypes may differ between studies using different populations.

**Figure 3:**
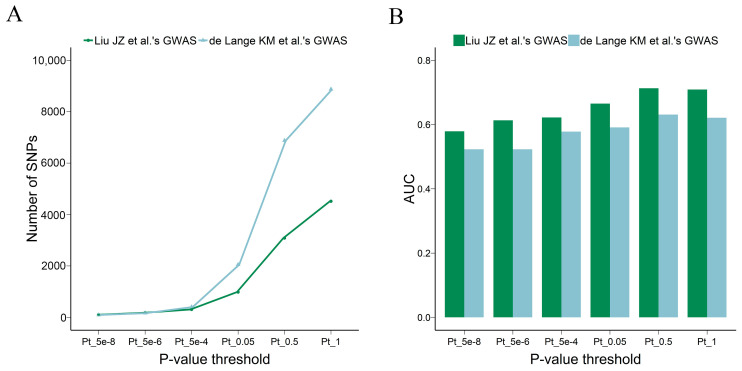
Effect of GWAS summary statistics on the power of the PRS. (**A**) shows the number of SNPs used to calculate PRS. (**B**) shows the AUC values of the PRS model. PRS: polygenic risk score, SNPs: single-nucleotide polymorphisms, GWAS: genome-wide association study, AUC: the area under the curve for the receiver operating characteristic curve [25,26].

For this reason, we compared two sets of GWAS summary statistics for UC, one from Liu JZ et al. and the other from de Lange KM et al. We used imputed and common SNPs to determine which set of GWAS summary statistics was more effective for the PRS model. Although the amount of SNP information provided by Liu JZ et al. was much smaller than that from de Lange KM et al. (Figure 3A), the predictive power of PRS using Liu JZ et al.’s data was significantly better than that using de Lange KM et al.’s data (Figure 3B), with an optimal AUC value of 0.713 at a *p*-value of 0.5 (Table S1).

### 3.4. The Contributions of Common and Low-Frequency SNPs to Predictions

**Figure 4:**
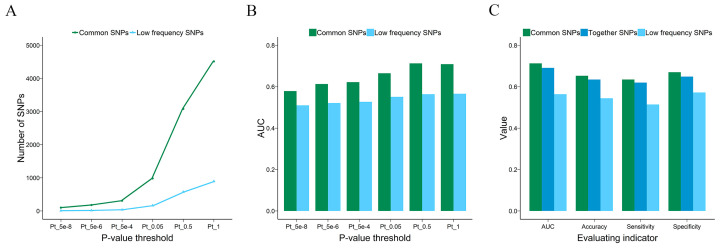
Effect of variant subsets on the power of the PRS. Line plot shows the number of SNPs used to calculate PRSs and histogram shows the AUC values of the PRS model. (**A**) shows the number of SNPs used to calculate PRS. (**B**) shows the AUC values of the PRS model. (**C**) shows the comparison results of PRS models based on SNPs at MAF > 0.05 (common), MAF ≥ 0.01 (together), or 0.05 ≥ MAF ≥ 0.01 (low-frequency). PRS: polygenic risk score, SNPs: single-nucleotide polymorphisms, AUC: the area under the curve for the receiver operating characteristic curve, MAF: minor allele frequency.

Based on minor allele frequency cutoffs of 0.01 and 0.05, SNPs are divided into rare, low-frequency and common categories [29]. GWAS are typically powered to characterize variants of MAF ≥ 0.01 and do not include the contributions from rare variants (MAF < 0.01). Therefore, we only analyzed common SNPs (MAF > 0.05) and low-frequency SNPs (0.05 ≥ MAF ≥ 0.01) from the imputed genetic data. Based on Liu JZ et al.’s GWAS summary statistics, we investigated the impact of using common and low-frequency SNPs on the predictive ability of the PRS model. The number of low-frequency SNPs used for PRS calculation was much less than common SNPs (Figure 4A), the PRS_lowfrequency_ models were all less powerful than the PRS_common_ models (Figure 4B), and the best AUC value for PRS_lowfrequency_ was only 0.566 (Table S1).

We further combined common and low-frequency SNPs and then calculated the PRS on the SNP set of MAF ≥ 0.01 by using Liu JZ et al.’s GWAS summary statistics and a *p*-value of 0.5. The resulting PRS model exhibited improved performance compared to the PRS model based solely on low-frequency SNPs, yet it was still less effective than the PRS model using only common SNPs (Figure 4C).

### 3.5. Population Stratification

**Figure 5:**
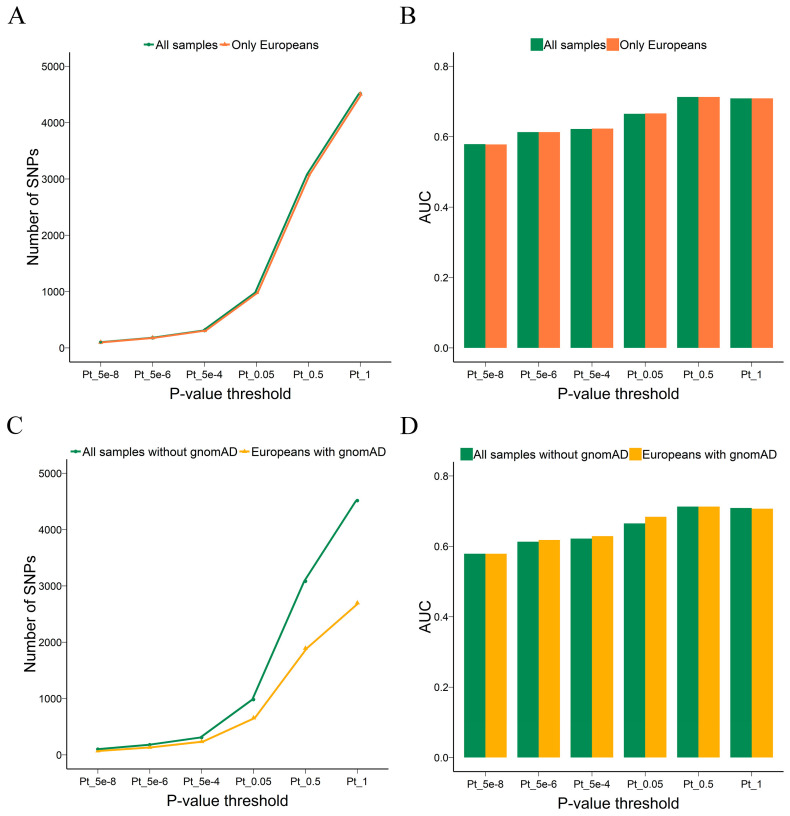
Effect of population stratification on the power of the PRS. Line plot shows the number of SNPs used to calculate PRSs and histogram shows the AUC values of the PRS model. (**A**,**B**) shows the results when comparing different sample sets for the PRS calculation. (**C**,**D**) shows the results when comparing all samples without selecting population-enriched sites and only European samples with population-enriched sites. PRS: polygenic risk score, SNPs: single-nucleotide polymorphisms, AUC: the area under the curve for the receiver operating characteristic curve.

As is known, population structure can be a confounding factor in PRS and different populations may have specific predispositions to diseases. For example, certain SNP loci showed association with UC in the Asian population but not in the European population [30]. Therefore, controlling the population stratification in the target dataset is an essential step in PRS. In this study, we trained the PRS model on genetically identified European samples only. We also evaluated how population stratification biases influence the PRS in UC; however, we did not observe inflations or deflations due to population structure, likely due to the small fraction of non-European samples in the target dataset. As shown in Figure 5A, the difference in the number of common SNPs used for PRS calculation was very small, and no improvement was found in the discriminative ability when the non-European samples were removed from cases and controls (Figure 5B).

In addition to removing non-European samples from this study, we further refined our analysis by screening for population-enriched SNPs. We intersected the European population-enriched sites selected from the gnomAD database with our data and used them for PRS calculation. Under the condition that only the population-enriched SNPs were reserved, the number of SNPs used for PRS calculation was greatly reduced under the same *p*-value threshold (Figure 5C), but the performance of the PRS model was enhanced at *p*-value thresholds of 5 × 10^−6^, 5 × 10^−4^, and 0.05, and the AUC value only decreased by 0.002 at the *p*-values of 1 (Figure 5D and Table S1). The results indicated that selecting population-enriched sites contributed to the PRS model, which seemingly filters out variants that are not informative, and the performance of the models was maintained with an average reduction of 32% of variants.

### 3.6. Performance Verification of the Best PRS

Considering all PRS models, we found that by using a genetically identified European population, the scores calculated on imputed, common, and population-enriched SNPs, as well as GWAS summary statistics from Liu JZ et al. with a *p*-value threshold of 0.5, had the best performance. This PRS was significantly associated with susceptibility to UC, with an AUC value of 0.713, corresponding to an accuracy of 0.660, a sensitivity of 0.626, and a specificity of 0.692. Furthermore, this PRS exhibits superior performance in disease status prediction, with both PPV and NPV surpassing those of other PRS models. Specifically, this model achieves a PPV of 0.656 and an NPV of 0.664, indicating relatively high accuracy in predicting the presence or absence of disease (Table S1).

**Figure 6:**
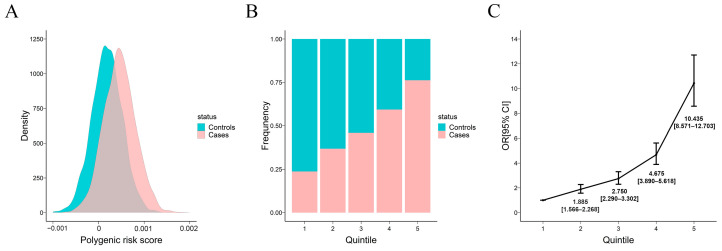
Distribution of the best PRS model and its association with UC. (**A**) Density of UC PRS in cases and controls. (**B**) Distribution of UC cases and controls in each quintile. (**C**) Disease OR for the second to fifth quintiles of the UC-PRS distribution among controls (first quintile used as reference). Vertical bars demarcate 95% confidence intervals. UC: ulcerative colitis, PRS: polygenic risk score, OR: odds ratio.

We plotted the kernel density estimates of the predicted risk scores for the control and case groups based on the best-performing PRS model (Figure 6A). There was a substantial separation of the cases from the controls. As expected from the AUC, the separation was profound for UC.

In order to measure the usefulness of the best PRS model, individual polygenic risk scores were ranked from lowest to highest and stratified into quintiles. We calculated the OR of case–control status by comparing each quintile to the lowest quintile as a reference. In the PRS quintiles, the distribution of UC cases increased from the lowest to the highest quintiles. Conversely, for the controls, the proportions showed the opposite pattern (Figure 6B). Notably, the OR was largest for the difference between the first and the fifth quintiles, and the OR for UC between the highest and lowest quintiles was 10.435 (95% CI 8.571–12.703) (Figure 6C). This finding is consistent with previous studies, further confirming that individuals at the tails of the PRS distribution can face significantly higher disease risk than the general population [6,9,31].

## 4. Discussion

In this study, we performed a step-by-step PRS model construction to predict an individual’s susceptibility to UC. The PRS, widely recognized for its effectiveness, is increasingly utilized in genetic risk prediction, potentially aiding clinical decision-making and early prevention. We employed cutting-edge approaches to improve the performance of PRS in terms of prediction of risk for UC. In addition, we comprehensively discussed the variables potentially affecting a PRS model, such as selecting better GWAS summary statistics, considering the population difference in imputation reference panels and the genetic background of the targeting individuals. After harmonizing the factors influencing the PRS across the whole PRS establishment procedures, our best PRS model performed well in UC risk prediction, reaching an AUC value of 0.713, which is comparable to other PRS analyses on UC and IBD [32,33,34]. Furthermore, the analyses on quintiles of PRS demonstrated the ability of the best PRS to discriminate between clinically relevant low-risk and high-risk groups.

A major strength of this study was that we fully considered the impact of genetic ancestry on all aspects of generating the PRS. Given that ancestry was a confounder or an independent predictor of many diseases, we used a novel approach to select the SNPs to calculate population-specific PRS. The removal of non-European samples had little effect on the results, which may be due to the relatively low proportion (4.0% non-European cases, 1.0% non-European controls, 2.5% non-European individuals in all samples) of non-European individuals in our samples. The impact on the results should be verified as this proportion increases. The effect of the population can be at the sample level or at the mutation level, as allele frequencies, LD patterns, and effect sizes of common SNPs vary with ancestry. Therefore, after removing non-European samples, we implemented an SNP filtration process to identify population-enriched variants for the European population. This refinement improved the performance of the PRS models at *p*-value thresholds of 5 × 10^−6^, 5 × 10^−4^, and 0.05. This demonstrated that screening the population-enriched SNPs for PRS calculations enhances the performance of the model. Additionally, we used the population-specific reference panel to impute our array data and used the population-specific GWAS summary statistics to calculate PRS. When we compare the impact of the two GWAS summary statistics on PRS, the differences between the summary statistics from Liu JZ et al. and de Lange KM et al. are not only in the number of GWAS samples and the amount of SNP information provided but also, more importantly, in the difference in the population initially utilized for the GWAS. The samples of de Lange KM et al. were of mixed ancestries, in contrast to Liu JZ et al., who solely used a European population. The inclusion of a non-European population may have an impact on SNP weights, and thus reduce the performance of the PRS in individuals of European descent [35]. Although our data also contained a mix of ancestries, with mostly European samples and very few Asian and individuals of mixed ancestry, the effect value of SNP derived from de Lange KM et al.’s GWAS summary statistics may be more divergent from the true weights of our genetic data, resulting in the PRS of de Lange KM et al. being significantly worse than the PRS of Liu JZ et al. This demonstrated the importance of using GWAS summary statistics that closely match the demographic structure of the target data when constructing PRS models.

Another strength of our study was that we thoroughly assessed the factors potentially affecting the capability of the PRS model. The effectiveness of imputation was verified by comparing the chip SNP subset’s prediction performance to that of the imputed SNPs. The imputed SNPs significantly increased the number of overlapping SNPs between our data and UC GWAS summary statistics and brought a great increase in the number of SNPs which are available in the PRS calculation. Additionally, using more common SNPs, selecting large scale GWAS summary statistics, and relaxing the *p*-value cutoff demonstrated that the inclusion of more phenotype-associated SNPs, even not reaching genome-wide significance, positively contributes to the predictive model [36]. However, using more SNPs to calculate the PRS does not mean a better performance of the PRS model, which is also affected by the quality of the SNPs, exemplified by the lower performance of the calculated PRS after combining low-frequency SNPs with common SNPs compared to the performance of the PRS calculated by using common SNPs. Using more variants is the simple rule to improve the PRS model, which is in line with reported studies, but it does not always work as uninformative variants could also be included when relaxing the inclusion threshold of *p*-values.

Our study has several limitations. First, the majority of the samples in our study were European, which reduced the generalizability of our findings. High-quality genetic studies in non-European populations remain a priority to apply more accurate PRS analyses to other populations [37]. Second, our current PRS model focuses exclusively on SNPs selected for their statistical significance, potentially overlooking variants that, while not reaching statistical significance, may still possess substantial predictive utility [38]. Future enhancements should include exploring alternative methods for selecting predictors beyond mere statistical significance, which represents a promising direction for improving the model’s predictive accuracy. Third, UC is a complex multifactorial disorder caused by the interplay of both genetic and non-genetic risk factors [1]. The PRS model will be more effective in predicting high-risk individuals if it is supplemented with non-genetic factors, which is hard to access for most of individuals, as with most PRS studies.

Our results suggest that the best PRS has a predictive value for UC, but it has not reached the goal of clinical application, as evidenced by a PPV of 0.656 and an NPV of 0.664. This could be driven by the lower heritability of UC and thus the benefit from PRS is also lower [39]. Furthermore, we used the genotype data from UC cases and controls to analyze the conditions for obtaining the best PRS model and the selection methods of these conditions can be extended to the establishment of PRS models for other diseases.

## 5. Conclusions

In summary, although the PRS is not yet capable of diagnosing UC in clinical practice, it has demonstrated the ability to stratify individuals at high risk for UC. This ability is likely to be strengthened in the future by the addition of more individualized data, such as clinical diagnoses’ information, lifestyle data, and environmental exposure data. To establish a robust PRS model for UC using genotype array datasets, several critical steps are necessary: selecting an appropriate reference panel for imputation, considering the genetic background involved in the GWAS summary, selecting informative SNPs in both the GWAS summary statistic and the target individuals, and accounting for the ethnic background of the target samples. We have thoroughly assessed the significance of these factors through a detailed, step-by-step construction process of the PRS and advise adhering to the principle that using population-specific data as much as possible can benefit PRS’s capability, which is more powerful than picking SNPs by other means. Moreover, taking advantage of population-enriched SNPs may reduce redundant sites for PRS. This study provided a comparable UC risk prediction model as well as guidance on how to avoid pitfalls when developing PRS models for UC, which can be applied to other PRS studies of complex diseases with mixed populations.

## Figures and Tables

**Table 1:** Description of the number of samples.

Samples	Male	Female	*p* Value
All samples (N = 5657)			
Cases	1369	1412	0.691
Controls	1432	1444	
Only European population (N = 5515)			
Cases	1310	1359	0.619
Controls	1417	1429	

**Table 2:** Description of the number of SNPs.

SNPs	Cases	Controls	Intersection ^1^	
			MAF > 0.05	0.05 ≥ MAF ≥ 0.01
Post-QC chip SNPs	850,439	184,241	14,289	1141
Imputed SNPs (INFO ≥ 0.8) 1000G reference panel ^2^	20,056,409	14,012,798	371,612	49,500
Imputed SNPs (INFO ≥ 0.8) European-specific reference panel ^3^	64,275,950	58,734,094	419,735	69,216

^1^ Intersection represents the same SNPs between cases and controls. ^2^ 1000G reference panel means the 1KGP Phase 3 (version 5b) reference panel. ^3^ European-specific reference panel means the 503 European samples in 1KGP Phase 3 (version 5b) reference panel.

## Data Availability

The ulcerative colitis cases (EGAD00000000025) and controls (EGAD00010000250) dataset is available for request upon approval by the Wellcome Trust Case Control Consortium 2 (https://www.wtccc.org.uk/, accessed on 17 June 2020). UC GWAS summary statistics are available at NHGRI-EBI GWAS Catalog (https://www.ebi.ac.uk/gwas/downloads/summary-statistics, accessed on 10 June 2022). The gnomAD v2.1.1 dataset is available at the gnomAD database (http://www.gnomad-sg.org/, accessed on 20 March 2023). The 1KGP reference panel is available at the database (https://www.internationalgenome.org/, accessed on 9 November 2021).

## Supplementary Materials

The following supporting information can be downloaded at: https://www.mdpi.com/article/10.3390/genes15060684/s1, Figure S1: A principal component analysis plot; Figure S2: Comparison of SNP counts before and after imputation with different reference panels; Table S1: The prediction details of the different PRS models.
