# Supplementary material for: A Polygenic Risk Analysis for Identifying Ulcerative Colitis Patients with European Ancestry
Source: Genes (Basel). 2024 May 25;15(6):684. doi: 10.3390/genes15060684 (PMC11202467; doi:10.3390/genes15060684)
Supplement: Supplementary file 1 [file genes-15-00684-s001.zip › genes-3016139-supplementary.pdf]

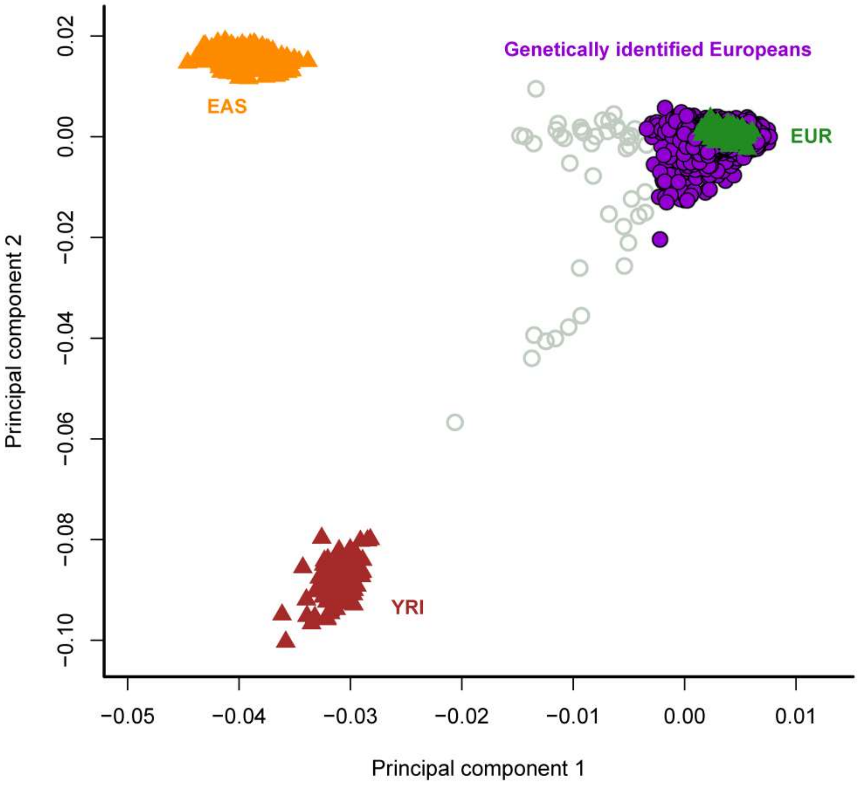

Figure S1. A principal component analysis plot. Samples projected on data of European ancestry (EUR), East Asian (EAS), Yoruba in Ibadan (YRI) from the 1000 Genomes Project database. The genetically identified Europeans were highlighted in purple.

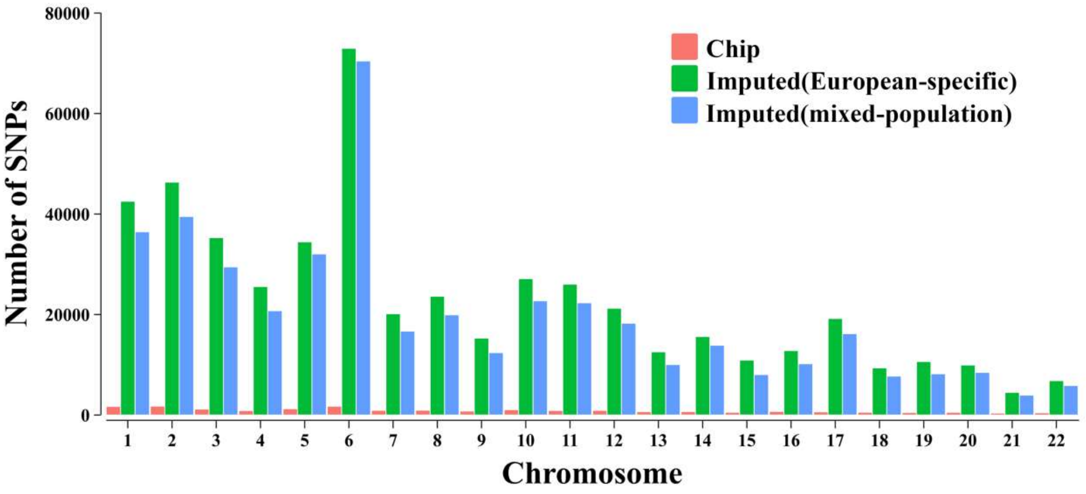

Figure S2. Comparison of SNP counts before and after imputation with different reference panels. European-specific means the 503 Europeans in 1000 Genomes Project Phase 3 (version 5b) reference panel, and mixed-population means the 1000 Genomes Project Phase 3 (version 5b) reference panel. SNPs: single nucleotide polymorphisms.

Table S1. The prediction details of the different PRS models

Computing conditions of the PRS	*P*-value	SNP number	AUC	ACC	Sensitivity	Specificity	PPV	NPV
	5×10^{-8}	36	0.472	0.483	0.216	0.741	0.446	0.494
Sample: all samples	5×10^{-6}	55	0.475	0.484	0.234	0.730	0.452	0.495
GWAS summary statistics: de Lange KM *et al.*	5×10^{-4}	119	0.512	0.510	0.384	0.633	0.503	0.515
Imputation: No	0.05	491	0.504	0.503	0.359	0.642	0.492	0.509
SNPs: MAF > 0.05	0.5	1,838	0.505	0.504	0.366	0.637	0.494	0.510
	1	2,930	0.506	0.505	0.372	0.634	0.495	0.510
	5×10^{-8}	92	0.523	0.521	0.429	0.610	0.515	0.525
Sample: all samples	5×10^{-6}	165	0.523	0.518	0.425	0.608	0.512	0.522
GWAS summary statistics: de Lange KM *et al.*	5×10^{-4}	404	0.578	0.554	0.511	0.596	0.550	0.558
Imputation: European reference panel	0.05	2,049	0.591	0.564	0.530	0.596	0.559	0.567
SNPs: MAF > 0.05	0.5	6,877	0.631	0.592	0.576	0.607	0.587	0.597
	1	8,875	0.621	0.587	0.568	0.604	0.581	0.591
	5×10^{-8}	90	0.555	0.538	0.473	0.600	0.533	0.541
Sample: all samples	5×10^{-6}	164	0.527	0.521	0.430	0.610	0.516	0.525
GWAS summary statistics: de Lange KM *et al.*	5×10^{-4}	388	0.524	0.519	0.424	0.611	0.513	0.523
Imputation: mixed-population reference panel	0.05	1,904	0.528	0.522	0.438	0.603	0.516	0.526
SNPs: MAF > 0.05	0.5	6,556	0.488	0.495	0.336	0.648	0.480	0.502
	1	8,554	0.457	0.478	0.223	0.724	0.438	0.491
	5×10^{-8}	99	0.579	0.557	0.516	0.597	0.553	0.560
Sample: all samples	5×10^{-6}	178	0.613	0.579	0.553	0.604	0.574	0.583
GWAS summary statistics: Liu JZ *et al.*	5×10^{-4}	308	0.622	0.586	0.562	0.611	0.582	0.590
Imputation: European reference panel	0.05	982	0.665	0.616	0.594	0.637	0.613	0.619
SNPs: MAF > 0.05	0.5	3,083	0.713	0.653	0.635	0.670	0.650	0.655
	1	4,515	0.709	0.649	0.629	0.669	0.647	0.651
	5×10^{-8}	8	0.510	0.506	0.354	0.653	0.496	0.511
Sample: all samples	5×10^{-6}	15	0.521	0.512	0.472	0.550	0.504	0.519
GWAS summary statistics: Liu JZ *et al.*	5×10^{-4}	37	0.527	0.519	0.484	0.552	0.511	0.525
Imputation: European reference panel	0.05	163	0.551	0.538	0.509	0.565	0.531	0.544
SNPs: 0.05 ≥ MAF ≥ 0.01	0.5	574	0.564	0.544	0.514	0.572	0.537	0.549
	1	895	0.566	0.544	0.514	0.573	0.538	0.550
Sample: all samples<br>GWAS summary statistics: Liu JZ *et al.*<br>Imputation: European reference panel<br>SNPs: MAF ≥ 0.01	0.5	3,657	0.691	0.635	0.620	0.649	0.631	0.639
	5×10^{-8}	99	0.578	0.557	0.475	0.634	0.549	0.563
Sample: only Europeans	5×10^{-6}	179	0.613	0.581	0.525	0.633	0.573	0.587
GWAS summary statistics: Liu JZ *et al.*	5×10^{-4}	308	0.623	0.587	0.533	0.638	0.580	0.593
Imputation: European reference panel	0.05	981	0.666	0.617	0.578	0.654	0.610	0.623
SNPs: MAF > 0.05	0.5	3,078	0.713	0.653	0.620	0.685	0.648	0.658
	1	4,510	0.709	0.650	0.615	0.683	0.646	0.654

Table S1. The prediction details of the different PRS models (Continued)

Computing conditions of the PRS	*P*-value	SNP number	AUC	ACC	Sensitivity	Specificity	PPV	NPV
	5×10^{-8}	70	0.579	0.554	0.472	0.631	0.545	0.560
Sample: only Europeans	5×10^{-6}	133	0.618	0.587	0.527	0.642	0.580	0.592
GWAS summary statistics: Liu JZ *et al.*	5×10^{-4}	235	0.629	0.595	0.542	0.645	0.589	0.600
Imputation: European reference panel	0.05	655	0.684	0.632	0.597	0.666	0.626	0.638
SNPs: MAF > 0.05; population-enriched sites	**0.5**	**1,892**	**0.713**	**0.660**	**0.626**	**0.692**	**0.656**	**0.664**
	1	2,698	0.707	0.656	0.623	0.687	0.651	0.660
